# Work Group Climate and Behavioral Responses to Psychological Contract Breach

**DOI:** 10.3389/fpsyg.2019.00067

**Published:** 2019-02-01

**Authors:** Yimo Shen, John M. Schaubroeck, Lei Zhao, Lei Wu

**Affiliations:** ^1^School of Psychology, Southwest University, Chongqing, China; ^2^Department of Management, Eli Broad College of Business and Department of Psychology, Michigan State University, East Lansing, MI, United States; ^3^Department of Fundamental Courses Teaching, Chongqing Youth Vocational and Technical College, Chongqing, China

**Keywords:** psychological contract breach, power distance climate, procedural justice climate, job performance, organizational citisenship behavior

## Abstract

Drawing on theories of social exchange and social information processing, we examined whether the influence of psychological contract breach on in-role performance and organization-directed citizenship behavior (OCBO) depends on work group climate levels, specifically procedural justice climate and power distance climate. The findings supported our hypothesis that psychological contract breach more strongly influences in-role performance and OCBO among members of units with favorable procedural justice climates. Support for a hypothesized moderating role of power distance climate was less conclusive. We discuss the implications of our model and findings for theories of employee–organization relationships and practice.

## Introduction

During the last three decades there has been a surge in interest in how and why employees respond in different ways when they perceive that others fail to meet obligations established in a social exchange relationship (Colquitt et al., 2013; van Dijke et al., 2018). The most salient social exchange relationship for many employees is with the organization itself. The construct of *psychological contract* refers to employees’ beliefs concerning the reciprocal obligations that exist between them and their employing organization (Rousseau, 1995; Morrison and Robinson, 1997). Scholars suggest that employees are increasingly prone to perceiving that their organizations failed to fulfill one or more of its implicit or explicit obligations to them as part of the employment relationship. Such *psychological contract breaches* (PCBs, hereafter) may often be due to the increased need for organizations to utilize idiosyncratic contractual arrangements that maintain flexibility in changing business environments (Robinson and Morrison, 2000). Nevertheless, very often such breaches precipitate within the employee intense feelings of violation and diminished trust in the organization (Deery et al., 2006). These reactions to PCB can produce a wide range of employee responses that are not organizationally desirable, such as turnover and absenteeism, counterproductive behaviors, reduced organizational citizenship behaviors (OCBs) or worsened in-role performance (Zhao et al., 2007).

Whereas failure to manage breach perceptions effectively may account for a considerable amount of turnover and counterproductive behavior, these reactions are risky and/or costly to individuals who pursue them and thus tend to be low in frequency. A more common response, yet also detrimental from the organization’s perspective, is for the employee perceiving breach to withhold his or her best performance and curtail extra-role behaviors that benefit the organization or group (Zhao et al., 2007; Henderson et al., 2008). The latter acts are conceptualized as OCBs. OCB refers to “individual behavior that is discretionary, not directly or explicitly recognized by the formal reward system, and that in the aggregate promotes the effective functioning of the organization” (Organ, 1988, p. 4). This paper examines how the group context, specifically the shared perceptions of members concerning procedural justice and power distance norms, influence the extent to which PCB discourages such behaviors. Characteristics of work groups may influence how employees respond when they perceive PCBs. Scholars have suggested that third parties such as peers play significant roles in how employees evaluate psychological contract fulfillment (Ho and Levesque, 2005). Technically, a contextual effect is observed when a shared context evaluated at a one level (e.g., the group) moderates a relationship between variables examined at a lower level (e.g., the individual; Hauser, 1974). We argue that it is important to understand the role of group context because context shapes how employees respond to events they interpret as breaching their implicit contract with the organization. Efforts to identify more suitable approaches for managing situations in which perceptions of breach arise will benefit from knowledge about how contextual variables may strengthen or weaken employees’ responses to PCB.

To date, however, investigations of psychological contract perceptions and outcomes that have incorporated context variables have tended to examine context as an individual rather than a shared perception (Turnley and Feldman, 1999; Rosen et al., 2009). When shared perceptions of a context factor have been examined (Epitropaki, 2013), context is tested as an antecedent of PCB rather than as a moderator variable. Thus, we sought to contribute to the PCB literature by specifying the theoretical roles of procedural justice climate and power distance climate as contextual variables that influence the strength of the relationship between individuals’ PCB and their OCBs and in-role work performance. We focus on the group context variables of procedural justice climate (Naumann and Bennett, 2000) and power distance climate (Yang et al., 2007), examining how they may moderate the relationship between PCB and in-role and extra-role behaviors. Procedural justice refers to the prevailing level of belief in a group that authorities apply fair procedures when they make allocation decisions and other judgments that are of concern to members (Naumann and Bennett, 2000; Erdogan and Bauer, 2010; Lin and Leung, 2014). In a group with a relatively high *procedural justice climate*, members share the belief that they are beneficiaries of a procedurally just system. We argue that whereas employees generally seek to reciprocate positively for a favorable procedural justice climate, a group context characterized by high procedural justice is likely to strengthen the bitterness of employees who perceive they are victims of PCB. This is because a norm for just treatment contrasts with their own experience, thus undermining the individual’s sense of being a valued group member. As a result, a favorable procedural justice climate may be associated with particularly strong negative relationships between PCB and desirable employee behaviors.

Group climates can also make certain norms more salient, as with a high power distance climate. *Power distance climate* refers to the extent members believe they should accept the decisions of authority figures. In a higher power distance group climate, members will feel constrained from engaging efforts to restore equity in the exchange relationship with the organization through negative reciprocity, as by reducing their performance or citizenship behaviors. We therefore argue that at higher levels of group power distance climate, there will be a weaker negative relationship between PCB and in-role and extra-role behaviors.

In sum, we sought to build theory about the role of group climate constructs in moderating the influence of PCBs on important behavioral outcomes. We argue that two group climate constructs, procedural justice climate and power distance climate, independently moderate relationships between PCB and performance and citizenship behaviors. We conducted a study at a pharmaceutical manufacturing company in China to test our model.

### Theoretical Framework

#### Influences of PCB on In-Role Performance and Organizational Citizenship Behavior

Because reciprocity norms (Gouldner, 1960) play a crucial role in determining how employees respond to their perceptions of broken promises, social exchange theory has served as a guiding framework for numerous studies of PCB (Hui et al., 2004a). When employees perceive that their employer has failed to fulfill promised obligations, in order to restore balanced in the exchange relationship they often retaliate by reducing their in-role behaviors as well as discretionary behaviors that are not specified in their formal job requirements (i.e., OCB; Lo and Aryee, 2003; Bal et al., 2010; Restubog et al., 2010). Such withdrawal of effort is seen to be motivated by a desire to restore balance in the exchange relationship (Chen et al., 2008). Conversely, when employees perceive the organization supports their own well-being and keeps its promises, they tend to exert positive effort to maintain a favorable exchange relationship, engaging in higher levels of in-role performance and OCB (Henderson et al., 2008).

Psychological contract breach exhibits reliable negative relationships with in-role performance (Lester et al., 2002; Turnley et al., 2003) and OCB (Lo and Aryee, 2003; Turnley et al., 2003; Restubog et al., 2007). Researchers distinguish between interpersonally directed OCBs (OCBIs), such as helping coworkers with their work, and organization-directed citizenship behavior (OCBO), such as representing the unit or organization at external functions. However, although an employee’s psychological contract may be influenced by leaders and other social agents, it is ultimately with the organization. Because employees’ dispositions toward their organizations are more likely to influence organization-directed rather than individual-directed citizenship behavior (Williams and Anderson, 1991; Cohen-Charash and Spector, 2001; Skarlicki and Latham, 2006), they may respond to PCB by reducing their OCBO. OCBIs are often directed toward peers and are therefore more subject to one’s perceived quality of social exchange relationships with peers (e.g., Peng et al., 2014), making them less theoretically related to PCB.

Based on the logic of social exchange theory, we first propose the following hypothesis:

Hypothesis 1(a,b): PCB is negatively related to in-role performance (H1a) and OCBO (H1b).

#### Contextual Moderators of PCB Influences on Behavior

A variety of theoretical perspectives emphasize how emergent states of work groups lead individuals to interpret and respond to their experiences and observations in different ways (Salancik and Pfeffer, 1978; Zohar and Hofmann, 2012). These perspectives suggest that descriptive and prescriptive social norms, as well as social cues provided by peers, shape group members’ causal attributions about events, direct their attention to particular features of the environment, and influence how they choose to respond to these attributions and interpretations. These emergent group states are reflected in group climate constructs. A facet of work unit climate exists when members develop shared reference points in understanding a topical domain, such as by agreeing about practices, norms, and values related to the topic (see Ostroff et al., 2003). Climate facets vary at different levels, ranging from work unit or group climates to organization climates. Because climate dimensions are shared perceptions of members, from a multilevel perspective they are normally conceptualized as composition constructs (Kozlowski and Klein, 2000).

Dimensions of climate have a powerful influence on behavior because they represent cues that are transmitted by multiple others in the individual’s work environment. Climate facets influence members’ behaviors because they inform them about whether specific behaviors will tend to produce particular consequences. Shared climates also call attention to aspects of the task and social environment that inform members about how they should behave (Zohar and Luria, 2005). Scholars suggest that climate mean levels are driven both by the *espousals* of leaders and by sense-making processes that evolve from individuals’ interactions with their peers and leaders (i.e., *enactment* processes). Enactment and espousal processes promote consensus in understandings among employees concerning beliefs about a domain (Zohar and Hofmann, 2012), such as the extent to which the organization supports and encourages learning (Cortini et al., 2016).

Employees formulate the expectations that constitute their psychological contracts based on communications from their managers and other organizational authorities. Very often, however, managers’ subsequent decisions precipitate perceptions of breach. Enactment processes are also critical, as other employees provide social comparison information and other cues that influence the judgments individuals make in calibrating their responses to breach perceptions. Through collective sense making processes (Weick, 1995), unit members learn what has “worked” for other members and how others make sense of events. This provides members with more confidence in selecting behavioral responses. For example, an employee may learn that it is not acceptable to his or her work peers to retaliate directly against a supervisor for actions the employee deems unfair. Enactment processes tend to be particularly dynamic when members perceive actions of the organization are not consonant with their espoused intentions and expectations (Levine and Moreland, 1987; Zohar and Luria, 2005).

Based on the dual roles of group climate dimensions in directing members’ interpretations about events and creating behavior-outcome expectations, we identified climate constructs that may be expected to moderate the relationship between PCB and work behaviors that are subject to negative reciprocity effects. Below we argue that the level of procedural justice climate affects whether individuals interpret PCB in a way that can lead them to attempt to restore equity in their social exchange relationship with the organization by withdrawing effort. In addition, the social information available to individuals from peers is affected by the group’s adherence to particular norms for behavior. Unlike procedural justice climate, power distance climate does not affect how individuals frame or interpret PCBs. Power distance climate (Yang et al., 2007) reflects normative pressures that constrain how individuals behave in response to PCBs. We suggest that a relatively high power distance climate may lead employees to perceive that withholding job or organization-directed contributions may come at a higher cost.

##### Procedural justice climate

Procedural justice climate is an aspect of the social context that may influence how individuals behave when they perceive a high level of PCB (Naumann and Bennett, 2000). Procedural justice refers to the fairness of the procedures used for making judgments about individuals that may affect their rewards and other outcomes (Thibaut and Walker, 1975; Leventhal, 1976). Procedural justice climate is a composition construct representing members’ shared perception of the prevailing level of procedural justice in their work unit or organization (Naumann and Bennett, 2000). PCB is distinct from procedural justice in that PCB refers to one’s evaluation of personal outcomes in relation to expectations transmitted to him or her in the course of the employment relationship, rather than an assessment of procedures or general fairness. Like the construct of distributive injustice, which refers to the perceived fairness in the distribution of rewards and other outcomes, PCB refers to individuals’ evaluations of their outcomes in the context of social exchange. However, employees’ assessments of personal outcomes in relation to their prior expectations established in contracting with the organization may bear little connection to whether they perceive their own outcomes as equitable. Psychological contracts are often idiosyncratic expectations of individual employees, many of which have little or no relation to broader justice principles. To illustrate, a newcomer may expect that he will be assigned to an office he was shown during the interview process. If that office is later assigned to a different employee and he receives a smaller office, he may perceive this as a psychological contract breach. Yet, the promised office may be larger than that of his peers at the same level, and a new policy ensures that when a larger office is available, it is offered to the senior-most peer. Thus, following through by directly remedying what the employee regards as a PCB would constitute a violation of what other employees regard as a practice that promotes procedural justice.

Individuals’ beliefs that link their fulfillment of their own specific obligations to their receipt of rewards are the basis of psychological contracts. When the mean level of procedural justice climate is high in the unit, employees share the perception that the formal systems under which they work tend to ensure that they will receive expected outcomes if they fulfill their obligations. Because procedural justice climate perceptions are shared within the work unit, individuals’ perceptions of procedural justice are reinforced in a group with a high procedural justice climate. Such a climate gives members more confidence in utilizing information about organizational procedures as a guide to action. A favorable procedural justice climate also helps to validate self-worth because it creates a common standard against which one may compare with peers how he or she is treated by authorities (Buckingham and Alicke, 2002). A high procedural justice climate mean level signals that the employer respects employees, and thus it can affirm positive expectations that each member’s psychological contract will be fulfilled (Tangirala and Ramanujam, 2008).

When individuals perceive a substantial degree of breach with respect to their own implicit expectations, however, a favorable procedural justice climate may make the breach more salient, precipitating stronger feelings of being betrayed by their employer (Morrison and Robinson, 1997; Elangovan and Shapiro, 1998). Thus, in this context we refer to PCB level in operational terms as the PCB level after centering by the group mean. Following the practice for other constructs such as LMX, we refer to group-mean centered PCB as *relative PCB* (e.g., Henderson et al., 2008). In this case, the individual is confronted with dissonant information that contrasts how the group is treated by the organization with how she herself is treated (Mayer et al., 2007). Employees in this situation (i.e., high relative PCB, high procedural justice climate) may tend to perceive they have been singled out for poor treatment. Such perceptions create feelings of relative deprivation (Crosby, 1976) that motivate negative reciprocity. As we noted above, studies suggest that employees often use their performance inputs as a means to redress what they perceive to be comparative inequities, particularly when these are associated with the perception that one is less respected or appreciated than others (e.g., Restubog et al., 2010). One such approach is to reduce the levels of one’s favorable contributions to the organization by reducing job performance and OCBO (e.g., Van Dijke et al., 2012; Janssen and Gao, 2015). Thus we predicted that the negative relationships between PCB and in-role performance and OCBO [Hypothesis 1(a,b)] are stronger when procedural justice climate is high.

When procedural justice climate is relatively low, employees do not expect outcomes will be administered through just processes, and accordingly a high PCB perception is less likely to lead an employee to believe he or she has been singled out for unjust treatment. Thus, a high procedural justice climate is a double-edged sword. It is desirable for establishing performance contingencies, yet when promises are for any reason perceived by the individual to have been broken, such a climate can lead to one to believe she is a less valued member as she did not receive fair treatment when others generally do. We therefore expected that in a low procedural justice climate, social comparisons with other members will have less influence on how employees evaluate their PCB and choose how to respond. When low expectations for procedural justice prevail in the group, PCB may be considered normal and employees may find it less threatening to their sense of status and belonging to the group and organization. Taking these arguments concerning high and low levels of procedural justice climate together leads us to the following hypothesis:

Hypothesis 2 (a,b): Procedural justice climate moderates the relationship between PCB and in-role performance (H2a) and OCBO (H2b), such that the relationship is more strongly negative when procedural justice climate is high compared to when it is low.

##### Power distance climate

Scholars of PCB have highlighted the role of shared values and culture in shaping perceptions of the employment relationship (Thomas et al., 2003; Hui et al., 2004b). Work groups develop distinct cultures, and their behavioral norms and shared values are the core defining elements of these cultures (Levine and Moreland, 1991). Groups’ values and norms represent their patterned approach to responding to a range of contingencies, reducing uncertainty about appropriate interpersonal behavior in the group and enabling more efficient member coordination (Feldman, 1984). Thus, work groups provide a context in which certain social values are likely to prevail. This provides cues members utilize in deciding how to respond to PCB. Such social cues aid members in making sense of the potential personal consequences on their reactions (Aquino et al., 2006). In response to unexpected events, we argue that employees tend to draw from the norms of their groups in deciding whether they should accept the breach as a prerogative of management or respond to it through acts of negative reciprocity.

Power distance climate (PDC, hereafter) has been conceptualized either as an antecedent or moderator variable in numerous studies (Earley, 1999; Schaubroeck et al., 2007; Yang et al., 2007; Cole et al., 2013; Vidyarthi et al., 2014). PDC refers to group level shared perceptions among group members about the extent they should defer to authorities (Yang et al., 2007). PDC may be important to how individuals respond to PCB because it provides cues employees use to evaluate whether particular responses to PCB are likely to be accepted by other members. In high PDC groups, there is a normative expectation that members will accept the prerogatives of the group leader (Yang et al., 2007). There will be social role pressures in high PDC groups for individuals to defer to the judgments of authority figures even when they may perceive that such judgments violate their psychological contracts with the organization. In such instances, peers may expect one should not respond behaviorally if one perceives a breach. Reducing one’s level of in-role performance or OCBO in response to PCB would be unacceptable and reflect unfavorably on the group. Conversely, under weak power distance norms employees regard themselves not merely as agents of authorities but as unique contributors who must be respected. Such respect is conveyed by being treated in a manner that is consistent with one’s expectations (Tyler and Blader, 2000). Thus, we expected that in response to PCB employees in high PDC groups would be more likely to seek to restore balance in their social exchange with the employer by reducing their in-role performance and OCBO than members of low PDC groups.

Hypothesis 3: Group power distance climate moderates the relationship between PCB and in-role performance (H3a) and OCBO (H3b), such that the relationship is more strongly negative when power distance climate is low compared to when it is high.

## Materials and Methods

We administered surveys to 312 employees and 86 supervisors of a pharmaceutical manufacturing company in China. Each work group was supervised by a single formal group leader, who was not a member of the group itself. Some respondents were excluded from the analyses for any of the following three reasons: They alternately lacked complete supervisor-reported evaluations of their in-role performance and/or OCBO, did not provide complete self-report data on key variables, or could not be matched to a unique work group. Additionally, some groups were excluded from the analyses if the number of members responding comprised fewer than three members. The final sample consisted of 232 subordinates and 71 supervisors, with an average of 3.26 members per group. A majority of non-supervisory participants were males (51%), relatively young (56% were 20–29 years of age), well educated (63% had completed specialty education or above), and 64% had worked for the organization for between 1 and 3 years.

This study was carried out in accordance with the recommendations of ‘Ethic of guidelines, The Institutional Review Board of the Department of Psychology at Southwest University’ with written informed consent from all participants. The Institutional Review Board of the Department of Psychology at Southwest University approved the protocol for this study. Prior to administering the surveys, we asked each supervisor to list the names of his or her subordinates. After recruiting participants via email, participants provided informed consent and acknowledged that their participation was strictly voluntary and that their surveys would be kept confidential. Respondents completed the questionnaire during working hours. They were instructed to seal the completed questionnaires in an envelope we provided and to return them directly to the researchers on site.

In the subordinate survey questionnaire, the respondents were asked to provide their demographic information and to assess their own perceptions of PCB, group procedural justice climate, and power distance climate. The group leader of each employee rated each follower on in-role performance and OCBO. Respondents took on average about 15 min to complete the survey. Upon completion, they each received a high-quality pen as a token of appreciation.

### Measures

Survey questions used a six-point Likert-type scale (1 = strongly disagree to 6 = strongly agree), except for those noted below. The questionnaires items were translated from the original English versions (except for Power Distance Climate) into Chinese by a professional translator. The first author then followed Brislin (1980) back-translation procedure to assure equivalence of the measures in the Chinese and the English versions.

### Psychological Contract Breach

We measured psychological contract breach using the five-item scale developed by (Robinson and Morrison, 2000). A sample item is “Almost all the promises made by my employer during recruitment have been kept so far” (reverse-scored). Cronbach’s alpha for this scale was 0.80.

### Procedural Justice Climate

Following Mossholder et al. (1998), we asked respondents to reflect on their overall evaluations of the procedural fairness of performance appraisal, raises, benefits, and work condition, the four facets central in the organizational management system. Two sample items from the four included in the scale are “Overall, the procedures and policies used by your organization to handle performance appraisal in your work unit are fair,” and “Overall, the procedures and policies your organization uses to determine working condition (e.g., workload, assignment, etc.) for the members in your work unit are fair.” We adapted the items by using a referent shift consensus model (Chan, 1998; Kozlowski and Klein, 2000), asking respondents to indicate the extent to which their group members would in general tend to agree with the statements. With items assessed at the group level, the alpha for this scale was 0.90. The mean *r*_*wg*(*j*)_ was 0.90, indicating substantial member agreement.

### Power Distance Climate

We adapted a scale developed by Yang et al. (1989) to measure power distance climate. Scholars have suggested that Chinese traditional values of respect for authority represent high power distance (Zhang et al., 2006; Leung, 2008). Sample items are “When people are in dispute, they should ask the most senior person to decide who is right”; and “The best way to avoid mistakes is to follow the instructions of senior persons.” To adapt the measure to index group climate (i.e., PDC), we shifted the referent of this measure by asking all respondents to indicate the extent to which their group tends to agree with the five statements by using the stem, “As a whole, people in my group feel that….” The internal consistency reliability estimate for PDC at the group level was 0.68. There was considerable agreement among members, with a mean *r*_*wg*(*j*)_ of 0.88.

### In-Role Performance

To measure in-role performance, supervisors completed a three-item scale by Van Scotter and Motowidlo (1996) for each of their direct reports. The scale assesses overall work performance (1 = very unsatisfactory; 7 = excellent). A sample item is “In comparison to others of the same rank, what do you [the supervisor] think of his or her work performance?” Cronbach’s alpha for this scale was 0.93.

### Organization-Directed Citizenship Behaviors

Organization-directed citizenship behaviors were assessed using a seven-item scale adapted from Williams and Anderson (1991). Two sample items are “Conserves and protects organizational property,” and “Attendance at work is above the norm.” Cronbach’s alpha for this scale was 0.82.

### Control Variables

Age, gender, education, and tenure with the organization were controlled for in our analyses. Older employees tend to report lower levels of PCB (Bordia et al., 2008). Whereas age tends to be related to performance and OCB, the direction of the relationship varies across studies. Whereas gender is not reliably related to performance or OCBO, men tend to report higher levels of PCB (Deery et al., 2006). Women are also seen to respond more favorably to procedural justice than men (Tyler, 1994; Johnson et al., 2006). Age, gender, education, and tenure with the organization have been included as control variables in previous PCB studies (e.g., Rousseau, 1995; Lo and Aryee, 2003; Restubog et al., 2010), and therefore including these variables enhances the comparability of our findings with prior work. Following previous research (e.g., Parker et al., 1995; Farh et al., 2007), age was divided into three categories: 20–29, 30–39, over 40 (years). Education had three categories: high-school education, specialty education, undergraduate education or above. Organizational tenure had five categories: less than 1 year, 1–3, 4–6, 7–9, over 10 years.

## Results

### Confirmatory Factor Analyses (CFA)

Prior to testing our hypotheses, we conducted confirmatory factor analyses using LISREL 8.70 (Jöreskog and Sörbom, 2004) on the focal variables at the individual level. As shown in Table 1, the congeneric measurement model, with five factors as defined by the instruments, exhibited superior fit to plausible alternative measurement models. We therefore proceeded to examine the aggregation statistics for Procedural Justice Climate and Power Distance Climate.

**Table 1:** Comparison of measurement models.

Model	Factors	*χ*^2^	*df*	NNFI	CFI	RMSEA	Model comparison test		
							comparison	*χ*Δ^*2*^	Δ*df*
Model 1: (Baseline model)	Five factors	606.38	242	0.89	0.91	0.08			
Model 2	Four factors; based on Model 1, items measuring in-role performance and OCBO combined into one factor	800.27	246	0.85	0.87	0.10	2 vs. 1	193.89^∗∗^	4
Model 3	Four factors; based on Model 1, items measuring procedural justice and power distance climate combined into one factor	733.48	246	0.86	88	0.09	3 vs. 1	127.10^∗∗^	4
Model 4	One factor; items measuring all five factors combined into one factor	2182.31	252	0.60	0.64	0.18	4 vs. 1	1575.93^∗∗^	10

*N = 232 (supervisor–subordinate dyads); ^∗∗^p < 0.01. OCBO, organization-directed citizenship behavior; NNFI, Non-Normed Fit Index; CFI, Comparative Fit Index; RMSEA, Root Mean Square Error of Approximation.*

### Aggregation

To evaluate the viability of aggregating the individual-level data on Procedural Justice Climate and Power Distance Climate to the group-level, we examined both between-groups variation and within-groups agreement (Bliese, 2000; Hofmann, 2002). Following Bliese (2000), we used ICC(1) to estimates the proportion of the total variance of a measure that is explained by group membership, and ICC(2) to assess the degree to which the group means within a sample are reliable. We also conducted *F* tests to ascertain whether between-group differences in the mean levels of the group-level scores are significant. Together these statistics supported a decision to aggregate both the Procedural Justice Climate responses [ICC(1) = 0.35, ICC(2) = 0.64; *F*(70,161) = 2.76, *p* < 0.001] and the Power Distance Climate responses [ICC(1) = 0.25, ICC(2) = 0.49; *F*(70,161) = 2.08, *p* < 0.001] to the group level (James et al., 1984; Bliese, 2000; Liao et al., 2010). Agreement within groups was assessed by calculating *r*_*wg*(*j*)_. As noted above (Measures), *r*_*wg*(*j*)_ was high for both Procedural Justice Climate (0.90) and Power Distance Climate (0.88).

### Descriptive Statistics and Correlations

The means, standard deviations, correlations among the variables are summarized in Table 2. The directions and magnitudes of the correlations are consistent with previous research. Given the nested structure of our data, one should be careful when interpreting the correlations with the group context variables (i.e., Procedural Justice Climate and Power Distance Climate). The hierarchical linear modeling results reported below (“Tests of Hypotheses”) provide more accurate estimates of the hypothesized relationships (Chen et al., 2012). Notably, the test statistics showed no evidence skewness or kurtosis in PCB [skewness = 0.09 (*SE* = 0.16), ns; kurtosis = −0.05 (*SE* = 0.32), *ns*], Procedural Justice Climate [skewness = −0.66 (*SE* = 0.29), *ns*; kurtosis = −0.09 (*SE* = 0.56), *ns*], or Power Distance Climate [skewness = −0.11 (*SE* = 0.29), *ns*; kurtosis = −0.38 (SE = 0.56), *ns*].

**Table 2:** Means, standard deviations, and correlations between variables.

	*M*	*SD*	1	2	3	4	5	6	7	8	9
**Level 1: individual-level**											
1. Age	1.49	0.50	–								
2. Gender	1.55	0.70	0.05	–							
3. Education	2.15	1.15	0.12	0.21^∗∗^	–						
4. Tenure with firm	2.32	1.20	−0.17^∗∗^	0.47^∗∗^	0.02	–					
5. Psychological contract breach	2.76	0.77	0.05	−0.03	−0.02	0.11	(0.80)				
6. In-role performance	4.92	1.06	0.07	0.01	0.02	0.12	−0.22^∗∗^	(0.93)			
7. OCBO	4.41	0.68	0.03	0.10	0.04	0.06	−0.23^∗∗^	0.53^∗∗^	(0.82)		
**Level 2: group-level**											
8. Power distance climate	3.13	0.52	(0.68)								
9. Procedural justice climate	4.18	0.63	0.22	(0.90)							

*N = 232 at Level 1; N = 71 at Level 2. Reliability coefficients for multi-item scales are shown in parentheses on the diagonal. OCBO, organization-directed citizenship behavior. ^∗^p < 0.05, ^∗∗^p < 0.01.*

### Tests of Hypotheses

Because individuals were nested within groups and we hypothesized cross-level relationships, we tested the hypotheses using HLM 6.06 (Raudenbush et al., 2004). To test Hypothesis 1, we grand mean centered the Level 1 variables, whereas we examined the cross-level interactions (Hypotheses 2 and 3) using group-mean centering (Hofmann and Gavin, 1998; Enders and Tofighi, 2007). Tests of null models confirmed that there was significant variance across groups with respect to both In-Role Performance and OCBO. For In-Role Performance, as indicated by the rather high ICC(1) value of 0.20, 20% of the variability in individual In-Role Performance can be attributed to the groups [χ^2^(70, *N*_group_ = 71; *N*_individual_ = 232) = 128.40, *p* < 0.001]. For OCBO, 53% of the variability in individual OCBO can be attributed to the groups [χ^2^(70, *N*_group_ = 71; *N*_individual_ = 232) = 312.66, *p* < 0.001]. Thus, there is substantial variability at the group level. This warrants testing the hypotheses using HLM.

Hypothesis 1 states that PCB is negatively related to In-Role Performance (H1a) and OCBO (H1b). The HLM results shown in Table 3 support this hypothesis (see Model 1). In support of the hypothesis, PCB was significantly and negatively related to In-Role Performance (γ = −0.35, *p* < 0.01) and OCBO (γ = −0.17, *p* < 0.01).

**Table 3:** Results of hierarchical linear modeling analyses for in-role performance and organization-directed citizenship behavior.

Variable	In-role performance				Organization-directed citizenship behavior			
	Model 1 ^a^	Model 2 ^a^	Model 3 ^b^	Model 4 ^b^	Model 1 ^a^	Model 2 ^a^	Model 3 ^b^	Model 4 ^b^
1. Intercept	4.32^∗∗^ (0.27)	4.32^∗∗^ (0.26)	4.31^∗∗^ (0.27)	4.31^∗∗^ (0.28)	4.17^∗∗^ (0.18)	4.25^∗∗^ (0.18)	4.21^∗∗^ (0.18)	4.18^∗∗^ (0.22)
2. Level 1 variables								
Gender	0.21 (0.13)	0.24 (0.13)	0.23 (0.13)	0.23 (0.13)	0.08 (0.08)	0.08 (0.08)	0.09 (0.08)	0.14 (0.10)
Age	−0.15 (0.11)	−0.16 (0.11)	−0.11 (0.10)	−0.11 (0.10)	0.01 (0.07)	0.01 (0.05)	0.02 (0.05)	−0.01 (0.06)
Education	0.04 (0.06)	0.03 (0.06)	0.02 (0.05)	0.02 (0.06)	0.04 (0.03)	0.02 (0.03)	0.01 (0.03)	−0.01 (0.05)
Organization tenure	0.19^∗∗^ (0.06)	0.20^∗∗^ (0.06)	0.18^∗∗^ (0.06)	0.18^∗∗^ (0.06)	0.01 (0.04)	−0.01 (0.03)	−0.01 (0.03)	0.02 (0.04)
PCB	−0.35^∗∗^ (0.09)	−0.39^∗∗^ (0.11)	−0.40^∗∗^ (0.09)	−0.40^∗∗^ (0.09)	−0.17^∗∗^ (0.05)	−0.15^∗^ (0.07)	−0.16^∗^ (0.07)	−0.22^∗∗^ (0.08)
3. Level 2 variables								
PDC		0.31 (0.16)	0.29 (0.16)	0.29 (0.17)		0.07 (0.13)	0.04 (0.13)	0.06 (0.13)
PJC		0.16 (0.15)	0.18 (0.14)	0.17 (0.14)		0.09 (0.11)	0.12 (0.11)	0.22 (0.13)
4. Level 2 interaction								
PCB × PDC			0.58^∗∗^ (0.15)	0.58^∗∗^ (0.15)			0.17 (0.14)	0.15 (0.14)
PCB × PJC			−0.35^∗∗^ (0.12)	−0.35 (0.12)			−0.20^∗^ (0.08)	−0.20^∗^ (0.08)
PDC × PJC				−0.01 (0.28)				0.32 (0.19)
Pseudo *R*^2c^	0.09	0.09	0.16	0.16	0.06	0.11	0.14	0.14

*N = 232 at Level 1; N = 71 at Level 2. Entries are estimates of fixed effects with robust standard errors. ^∗^p < 0.05; ^∗∗^p < 0.01. PCB, psychological contract breach; PJC, procedural justice climate; PDC, power distance climate. ^a^Level 1 and Level 2 variables are grand mean centered. ^b^Level 1 variables are group mean centered. Level 2 variables are grand mean centered; ^c^Pseudo R^2^ is calculated according to proportional change of Level 1 and Level 2 error variance (Snijders and Bosker, 2011).*

**FIGURE 1:**
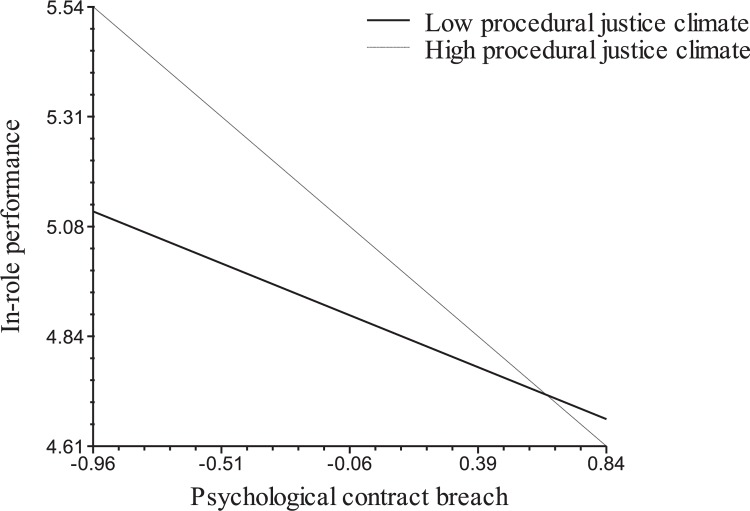
Cross-level moderating effects of procedural justice climate on the relationship between psychological contract breach and in-role performance. Simple slopes are –0.18 (*p* < 0.05) for lower procedural justice climate and –0.56 (*p* < 0.01) for higher procedural justice climate.

**FIGURE 2:**
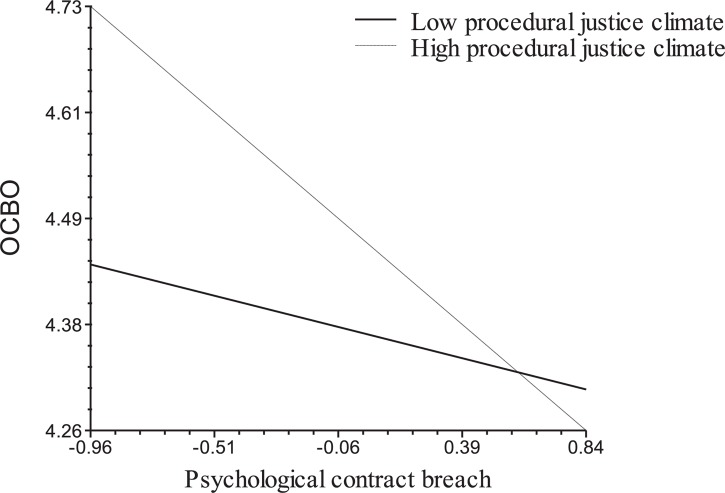
Cross-level moderating effects of procedural justice climate on relationship between PCB and organization-directed citizenship behavior (OCBO). Simple slopes are –0.03 (*ns*) for lower procedural justice climate and –0.29(*p* < 0.05) for higher procedural justice climate.

Hypothesis 2 states that the negative relationships between PCB and In-Role Performance (H2a) and OCBO (H2b) are moderated by Procedural Justice Climate, such that the relationships will be stronger when Procedural Justice Climate is relatively high. As shown for Model 3 in Table 3, the interaction between PCB and Procedural Justice Climate is significant in predicting In-Role Performance (γ = −0.35, *p* < 0.01) and OCBO (γ = −0.20, *p* < 0.05). We followed Aiken and West’s (Aiken and West, 1991) approach to examine the form of the interaction at two levels of Procedural Justice Climate, i.e., one standard deviation above and below the mean. We also computed the simple slopes at each of the two levels (Preacher et al., 2006). Figures 1, 2 show the patterns of the cross-level moderating effects of Procedural Justice Climate on the relationship between PCB and the outcomes of In-Role Performance and OCBO, respectively. Consistent with Hypothesis 2a, the simple slope tests reported and illustrated in Figure 1 indicate that PCB is negatively related to In-Role Performance at both the higher and the lower levels of Procedural Justice Climate, but the relationship between PCB and In-Role Performance is stronger when procedural justice climate is high. Similarly, as shown in Figure 2, simple slope tests indicate that PCB is not significantly related to OCBO when Procedural Justice Climate is lower, whereas it is negative and significant when Procedural Justice Climate is higher. Thus, Hypothesis 2 was supported.

**FIGURE 3:**
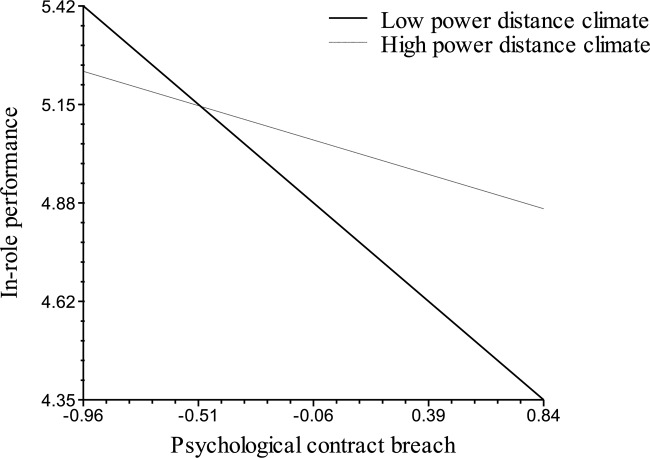
Cross-level moderating effects of power distance climate on relationship between PCB and in-role performance. Simple slopes are –0.65 (*p* < 0.01) for lower power distance climate and –0.10 (*ns*) for higher power distance climate.

Hypothesis 3 states that the negative relationships between PCB and the outcomes of In-Role Performance (H3a) and OCBO (H3b) are moderated by Power Distance Climate, such that the relationships will be weaker when Power Distance Climate is high. As shown for Model 3 in Table 3, the interaction of PCB and Power Distance Climate is significantly related to In-Role Performance (γ = 0.58, *p* < 0.01), but it is not significantly related to OCBO (γ = 0.17, *ns*). Figure 3 provides a graphical representation of the cross-level moderating effect of Power Distance Climate on the relationship between PCB and In-Role Performance, as well as the simple slope tests. These tests indicate that the relationship between PCB and In-Role Performance is weaker among groups with higher Power Distance Climate compared to groups with lower Power Distance Climate. There is a significant relationship between PCB and In-Role Performance only when Power Distance Climate is lower. Hypothesis 3a was therefore supported, whereas Hypothesis H3b was not supported.

In summary, except for the interaction between PCB and power distance climate predicting OCBO, the hypotheses were supported. One concern relates to the rather high amount of variance in OCBO scores that was attributable to group membership. One possible reason for the high group level homogeneity in OCBO values may relate to the content of the OCBO measure. Much of the item content in the Williams and Anderson (1991) OCBO index concerns organizational expectations for compliance. Rule-following is heavily influenced by normative pressures in work groups, even to the extent that a ‘rules and procedures climate’ construct has been proposed (Treviño et al., 1998; Elçi and Alpkan, 2009).

Yang et al. (2007) reported that procedural justice climate and power distance climate interacted in predicting organizational commitment and OCBO. Such an interaction would complicate the interpretation of our hypothesis tests. We therefore also tested these interactions. The findings show that the interactions of procedural justice climate and power distance climate were not significantly related to in-role performance or OCBO (see Model 4 of Table 3).

## Discussion

Organizational scholars have suggested that contextual factors play an important role in understanding how employees respond when they perceive that their expectations concerning the employment relationship have been violated (e.g., Rousseau and Schalk, 2000; Coyle-Shapiro and Shore, 2007; Dulac et al., 2008; Fitzsimmons and Stamper, 2014). However, little empirical and theoretical research has sought to clarify how organizational context affects employee reactions to PCB. We examined how group context variables, specifically procedural justice climate and power distance climate, influence how strongly individuals’ experiences of PCB affect their in-role performance and OCBO. This research responds to calls in recent years to examine the role of context in social exchange relationships, including psychological contracts in general and PCB in particular (Restubog et al., 2015). Our study extended the limited research on how group context influences individuals’ responses to PCB by group climate constructs. We found that PCB was more strongly related to in-role performance and OCBO among workers who were members of groups with favorable procedural justice climates. In addition, PCB was less strongly related to in-role performance among members of groups with norms that supported adherence to authority.

### Theoretical and Practical Implications

The strong support for Hypothesis 1, which stated that PCB has a significant negative influence on in-role performance and OCBO, is consistent with prior research indicating that employees often attempt to remedy a perceived imbalance of the social exchange relationship with another party that is not to their own favor by reducing their inputs to the relationship (Turnley et al., 2003). Whereas such negative reciprocity can take the form of engaging in negative acts such as deviant behavior (Barclay et al., 2005), employees tend to avoid these behaviors because engaging them incurs risks and may violate their own personal values. Employees’ negative reciprocity is more often expressed by reducing effort that is beneficial to the organization, such as by exhibiting lower levels of in-role performance and OCBO.

The primary focus of this research concerns the moderating effects of procedural justice climate and power distance climate were. The findings suggest that employee reactions to PCB are influenced by contextual cues related to how one should respond to adverse treatment. Our findings are consistent with Mayer et al. (2007) observation that a favorable procedural justice climate provides employees who experience injustice with social comparison information about the organization’s treatment of members. In the absence of PCB, individuals have more basis for a favorable exchange relationship when they perceive high justice is a norm for their group. Yet, particularly in groups in which most members maintain favorable perceptions of procedural justice, individuals who experience PCB may feel singled out for poor treatment, leading them to feel that they are less valued and respected. They may reconcile their (perceived) relative mistreatment by reducing their contributions to the organization. Because work unit peers normally serve as referents employees use to evaluate how the organiation fulfills its promises (Ho and Levesque, 2005), such a climate signals to the aggrieved member that he or she may be less respected and valued by organizational authorties than his or her peers, thereby further upending expectations for positive social exchange and eliciting negative reciprocity. Thus, PCBs have a greater negative impact on behaviors that reflect positive reciprocity (i.e., citizenship behaviors, higher in-role performance) when the work group climate is high on procedural justice because this climate suggests that the individual does not benefit from the fair treatment that is extended to other members.

Previous research on procedural justice climate has observed generally favorable influences on employee attitudes and behavior (Naumann and Bennett, 2000; Liao and Rupp, 2005). Our findings suggest social comparison processes may play a significant role in how employees respond to breach, particularly when the group perceived a favorable procedural justice climate. PCB tends to be a highly individualized phenomenon, such that breaches are not normally experienced by all members of the group simultaneously. This provides scope for the initiation of social comparison processes among individuals who perceive a breach. Employees who experience adverse outcomes are more prone to reflect on procedural justice (Brockner and Wiesenfeld, 1996; Folger and Cropanzano, 2001), and PCBs should be no exception. When noting that others in their group seem to perceive a favorable procedural justice climate, they may speculate as to why they experience breaches when others do not. When people perceive they have been singled out for poor treatment, this elicits the adverse emotional states associated with relative deprivation and motivates efforts to restore a sens of balance in the social exchange relationship by reducing inputs to the exchange. The least costly and risky means for most employees to do this is by lowering their effort levels.

The results concerning power distance climate were less conclusive than for procedural justice climate, as only one of the two interactions we tested was supported (i.e., the test of Hypothesis 3a, concerning in-role performance). Whereas there has been considerable emphasis in the organizational literature on individual, organizational, and societal values (Kirkman et al., 2006), there has been comparatively little attention to how values can be manifest at the work unit level and reflected in behavioral norms. Yet, group members’ proximity and interaction patterns make it likely that particular values become embodied in behavioral norms, leading to the formation and maintenance of distinct value orientations (Earley and Gibson, 1998; Yang et al., 2007). As we have argued, shared group values and norms affect how employees respond to PCB because they denote to members whether particular responses to events and experiences are appropriate. The values and norms of the group may constrain how individuals respond to their personal experiences of breach. Our findings suggest that among groups that maintained values that respected the prerogatives of authorities (i.e., high power distance climate), members were less likely to respond to breach by withholding performance effort. Members may expect little support from other group members for responding to PCB by withholding job-related effort. However, this moderating role of power distance climate did not extend to OCBO.

The current studies have important practical implications for managers, as they suggest that a carefully cultivated climate of high procedural justice can actually worsen how employees respond to perceptions of PCB. This does not imply that managers should prevent or inhibit the buildup of procedural justice, as the positive benefits of strong procedural justice climates are substantial. This is reflected in the significant and positive main effects of procedural justice climate on in-role performance and OCBO in our study. However, these findings underscore the importance for managers to address potential breaches, or perceptions of breaches, with affected employees directly, and behave in a manner consistent with procedural justice principles. In some cases, however, it is not merely poor information or a misunderstanding that created the perceived breach. In such instances, the perception of breach can undo a well-established basis of trust that is often hard to re-establish.

Organizations can potentially reduce PCBs by implementing more formalized and internally consistent human resource practices. Guzzo and Noonan (1994) noted that HRM practices are the primary means through which organizations communicate the expectations that create employees’ psychological contracts. Managers are less likely to communicate information that creates expectations that will later be unmet, and thus elicit breach perceptions, if job designs are matched with clear performance expectations and adequate employee training, employees are selected, socialized, evaluated and rewarded based on clear and consistent principles, and there are plans in place to ensure that high performers with potential for advancement have opportunities to do so (Bowen and Ostroff, 2004).

### Limitations and Future Research Directions

The cross-sectional, non-randomized design of our study precluded us from making definitive causal statements. However, our focus was on testing the cross-level moderating effects of procedural justice climate and power distance climate. Common method variance cannot produce spurious interaction effects (Farh et al., 2007). Nevertheless, field research on PCB could benefit from experimental or quasi-experimental designs to test causal relations between PCB and the various outcomes that have been the focus of this literature. Whereas manipulating PCB may not be practical, studies could bracket actual organizational events that affect procedural justice perceptions of employees in some units more than others (e.g., Greenberg, 1990) and examine whether such higher-level differences moderate the impact of PCB on outcomes.

The limited support for the hypothesized interactions involving power distance climate could possibly reflect the high power distance societal culture in which the study was conducted. Although there was considerable variance in power distance climate across groups, it may be expected that norms and values associated with high power distance are higher in China than in most societal settings outside of Asia and the Middle East. Societal culture in China has been strong influenced by Confucian ideology, which emphasizes behaving with deference to individuals of higher authority. Thus a form of ceiling effect pertaining to the moderating variable of power distance climate may have limited the potential to detect the hypothesized interaction effect. In future research, it will be useful to examine the effects of group power distance climate in moderating relationships between PCB and performance-oriented behaviors in different societal cultures that vary in their levels of average power distance. It will also be valuable to assess the role that team performance plays in this process. Shared beliefs can be particularly influential in higher performing groups (West, 2012). Future research may also benefit by considering the role of team members’ tenure in the team, as individuals may be more strongly influenced by group norms or values, such as are reflected in power distance climate, when they have longer standing relations with other team members.

## Conclusion

This study contributes to the literature by demonstrating that the group context, as reflected in group climate variables, substantially affects the strength of the relationship between PCB and behaviors that benefit the organization. PCB had an especially detrimental impact on employees’ behavior when they were members of groups with a high procedural justice climate or their groups adhered to norms and values that emphasize obedience to authority. These findings suggest that the group context conditions employees’ responses to PCB, both by enabling social comparison processes that affects how employees interpret their own experience of PCB and by creating expectations about the social consequences of different means to restore equity in one’s social exchange relationships with the organization.

## Author Contributions

YS, LZ, and LW contributed to collecting the study data and conducted the analysis. YS also contributed to the writing of the manuscript. JS contributed primarily to the conceptual development and manuscript writing.

## Conflict of Interest Statement

The authors declare that the research was conducted in the absence of any commercial or financial relationships that could be construed as a potential conflict of interest.

## References

[B1] Aiken L. S. West S. G. (1991). *Multiple Regression: Testing and Interpreting Interactions.* Newbury Park, CA: Sage Publications.

[B2] Aquino K. Tripp T. M. Bies R. J. (2006). Getting even or moving on? Power, procedural justice, and types of offense as predictors of revenge, forgiveness, reconciliation, and avoidance in organizations. *J. Appl. Psychol.* 91 653–668. 10.1037/0021-9010.91.3.653 16737361

[B3] Bal P. M. Chiaburu D. S. Jansen P. G. W. (2010). Psychological contract breach and work performance: is social exchange a buffer or an intensifier? *J. Manage. Psychol.* 25 252–273. 10.1108/02683941011023730

[B4] Barclay L. J. Skarlicki D. P. Pugh S. D. (2005). Exploring the role of emotions in injustice perceptions and retaliation. *J. Appl. Psychol.* 90 629–642. 10.1037/0021-9010.90.4.629 16060783

[B5] Bliese P. D. (2000). “Within-group agreement, non-independence, and reliability: implications for data aggregation and analysis,” in *Multilevel Theory, Research and Methods in Organizations*, eds Klein K. J. Kozlowski S. W. J. (San Francisco, CA: Jossey-Bass).

[B6] Bordia P. Restubog S. L. D. Tang R. L. (2008). When employees strike back: investigating mediating mechanisms between psychological contract breach and workplace deviance. *J. Appl. Psychol.* 93 1104–1117. 10.1037/0021-9010.93.5.1104 18808228

[B7] Bowen D. E. Ostroff C. (2004). Understanding HRM–firm performance linkages: the role of the “strength” of the HRM system. *Acad. Manage. Rev.* 29 203–221.

[B8] Brislin R. W. (1980). Translation and content analysis of oral and written material. *Handb. Cross-Cult. Psychol.* 2 349–444.

[B9] Brockner J. Wiesenfeld B. M. (1996). An integrative framework for explaining reactions to decisions: interactive effects of outcomes and procedures. *Psychol. Bull.* 120 189–208. 10.1037/0033-2909.120.2.189 8831296

[B10] Buckingham J. T. Alicke M. D. (2002). The influence of individual versus aggregate social comparison and the presence of others on self-evaluations. *J. Pers. Soc. Psychol.* 83 1117–1130. 10.1037/0022-3514.83.5.1117 12416916

[B11] Chan D. (1998). Functional relations among constructs in the same content domain at different levels of analysis: a typology of composition models. *J. Appl. Psychol.* 83 234–248. 10.1037/0021-9010.83.2.234

[B12] Chen X. P. Liu D. Portnoy R. (2012). A multilevel investigation of motivational cultural intelligence, organizational diversity climate, and cultural sales: evidence from US real estate firms. *J. Appl. Psychol.* 97 93–106. 10.1037/a0024697 21806296

[B13] Chen Z. X. Tsui A. S. Zhong L. (2008). Reactions to psychological contract breach: a dual perspective. *J. Organ. Behav.* 29 527–548. 10.1002/job.481

[B14] Cohen-Charash Y. Spector P. E. (2001). The role of justice in organizations: a meta-analysis. *Organ. Behav. Hum. Decis. Process.* 86 278–321. 10.1006/obhd.2001.2958

[B15] Cole M. S. Carter M. Z. Zhang Z. (2013). Leader-team congruence in power distance values and team effectiveness: the mediating role of procedural justice climate. *J. Appl. Psychol.* 98 962–973. 10.1037/a0034269 24060159

[B16] Colquitt J. A. Scott B. A. Rodell J. B. Long D. M. Zapata C. P. Conlon D. E. (2013). Justice at the millennium, a decade later: a meta-analytic test of social exchange and affect-based perspectives. *J. Appl. Psychol.* 98 199–236. 10.1037/a0031757 23458336

[B17] Cortini M. Pivetti M. andCervai S. (2016). Learning climate and job performance among health workers. A pilot study. *Front. Psychol.* 25:1644. 10.3389/fpsyg.2016.01644 27826274 PMC5078782

[B18] Coyle-Shapiro J. A. M. Shore L. M. (2007). The employee-organization relationship: where do we go from here? *Hum. Resour. Manage. Rev.* 17 166–179. 10.1016/j.hrmr.2007.03.008

[B19] Crosby F. (1976). A model of egoistical relative deprivation. *Psychol. Rev.* 83 85–113. 10.1037/0033-295X.83.2.85

[B20] Deery S. J. Iverson R. D. Walsh J. T. (2006). Toward a better understanding of psychological contract breach: a study of customer service employees. *J. Appl. Psychol.* 91 166–175. 10.1037/0021-9010.91.1.166 16435946

[B21] Dulac T. Coyle-Shapiro J. A. M. Henderson D. J. Wayne S. J. (2008). Not all responses to breach are the same: the interconnection of social exchange and psychological contract processes in organizations. *Acad. Manage. J.* 51 1079–1098. 10.5465/amj.2008.35732596

[B22] Earley P. C. (1999). Playing follow the leader: status-determining traits in relation to collective efficacy across cultures. *Organ. Behav. Hum. Decis. Process.* 80 192–212. 10.1006/obhd.1999.2863 10579962

[B23] Earley P. C. Gibson C. B. (1998). Taking stock in our progress on individualism-collectivism: 100 years of solidarity and community. *J. Manage.* 24 265–304. 10.1177/014920639802400302

[B24] Elangovan A. R. Shapiro D. L. (1998). Betrayal of trust in organizations. *Acad. Manage. Rev.* 23 547–566. 10.5465/amr.1998.926626

[B25] Elçi M. Alpkan L. (2009). The impact of perceived organizational ethical climate on work satisfaction. *J. Bus. Ethics* 84 297–311. 10.1007/s10551-008-9709-0

[B26] Enders C. K. Tofighi D. (2007). Centering predictor variables in cross-sectional multilevel models: a new look at an old issue. *Psychol. Methods* 12 121–138. 10.1037/1082-989X.12.2.121 17563168

[B27] Epitropaki O. (2013). A multi-level investigation of psychological contract breach and organizational identification through the lens of perceived organizational membership: testing a moderated-mediated model. *J. Organ. Behav.* 34 65–86. 10.1002/job.1793

[B28] Erdogan B. Bauer T. N. (2010). Differentiated leader–member exchanges: the buffering role of justice climate. *J. Appl. Psychol.* 95 1104–1120. 10.1037/a0020578 20718530

[B29] Farh J. L. Hackett R. D. Liang J. (2007). Individual-level cultural values as moderators of perceived organizational support-employee outcome relationships in China: comparing the effects of power distance and traditionality. *Acad. Manage. J.* 50 715–729. 10.5465/amj.2007.25530866

[B30] Feldman D. C. (1984). The development and enforcement of group norms. *Acad. Manage. Rev.* 9 47–53. 10.5465/amr.1984.4277934

[B31] Fitzsimmons S. R. Stamper C. L. (2014). How societal culture influences friction in the employee-organization relationship. *Hum. Resour. Manage. Rev.* 24 80–94. 10.1016/j.hrmr.2013.07.001

[B32] Folger R. Cropanzano R. (2001). Fairness theory: justice as accountability. *Adv. Organ. Just.* 1 1–55.

[B33] Gouldner A. W. (1960). The norm of reciprocity: a preliminary statement. *Am. Sociol. Rev.* 25 161–178. 10.2307/2092623

[B34] Greenberg J. (1990). Employee theft as a reaction to underpayment inequity: the hidden cost of pay cuts. *J. Appl. Psychol.* 75 561–568. 10.1037/0021-9010.75.5.561

[B35] Guzzo R. A. Noonan K. A. (1994). Human resource practices as communications and the psychological contract. *Hum. Resour. Manage.* 33 447–462. 10.1002/hrm.3930330311

[B36] Hauser R. M. (1974). Contextual analysis revisited. *Sociol. Methods Res.* 2 365–375. 10.1177/004912417400200305

[B37] Henderson D. J. Wayne S. J. Shore L. M. Bommer W. H. Tetrick L. E. (2008). Leader-member exchange, differentiation, and psychological contract fulfillment: a multilevel examination. *J. Appl. Psychol.* 93 1208–1219. 10.1037/a0012678 19025243

[B38] Ho V. T. Levesque L. L. (2005). With a little help from my friends (and substitutes): social referents and influence in psychological contract fulfillment. *Organ. Sci.* 16 275–289. 10.1287/orsc.1050.0121

[B39] Hofmann D. A. (2002). “Issues in multilevel research: theory development, measurement, and analysis,” in *Handbook of Research Methods in Industrial and Organizational Psychology*, ed. Rogelberg S. G. (Oxford: Blackwell), 247–274.

[B40] Hofmann D. A. Gavin M. B. (1998). Centering decisions in hierarchical linear models: implications for research in organizations. *J. Manage.* 24 623–641. 10.1177/014920639802400504

[B41] Hui C. Lee C. Rousseau D. M. (2004a). Employment relationships in China: do workers relate to the organization or to people? *Organ. Sci.* 15 232–240. 10.1287/orsc.1030.0050

[B42] Hui C. Lee C. Rousseau D. M. (2004b). Psychological contract and organizational citizenship behavior in China: investigating generalizability and instrumentality. *J. Appl. Psychol.* 89 311–321. 10.1037/0021-9010.89.2.311 15065977

[B43] James L. R. Demaree R. G. Wolf G. (1984). Estimating within-group interrater reliability with and without response bias. *J. Appl. Psychol.* 69 85–98. 10.1037/0021-9010.69.1.85

[B44] Janssen O. Gao L. (2015). Supervisory responsiveness and employee self-perceived status and voice behavior. *J. Manage.* 41 1854–1872. 10.1177/0149206312471386

[B45] Johnson R. E. Selenta C. Lord R. G. (2006). When organizational justice and the self-concept meet: consequences for the organization and its members. *Organ. Behav. Hum. Decis. Process.* 99 175–201. 10.1016/j.obhdp.2005.07.005

[B46] Jöreskog K. G. Sörbom D. (2004). *Interactive LISREL.* Skokie, IL: Scientific Software International. Inc.

[B47] Kirkman B. L. Lowe K. B. Gibson C. B. (2006). A quarter century of culture’s consequences: a review of empirical research incorporating Hofstede’s cultural values framework. *J. Int. Bus. Stud.* 37 285–320. 10.1057/palgrave.jibs.8400202

[B48] Kozlowski S. W. J. Klein K. J. (2000). “A multilevel approach to theory and research in organizations: Contextual, temporal, and emergent processes,” in *Multilevel Theory, Research, and Methods in Organizations: Foundations, Extensions, and New Directions*, eds Klein K. J. Kozlowski S. W. J. (San Francisco, CA: Jossey-Bass), 3–90.

[B49] Lester S. W. Turnley W. H. Bloodgood J. M. Bolino M. C. (2002). Not seeing eye to eye: differences in supervisor and subordinate perceptions of and attributions for psychological contract breach. *J. Organ. Behav.* 23 39–56. 10.1002/job.126

[B50] Leung K. (2008). Chinese culture, modernization, and international business. *Int. Bus. Rev.* 17 184–187. 10.1016/j.ibusrev.2008.02.009 11293040

[B51] Leventhal G. S. (1976). “The distribution of rewards and resources in groups and organizations,” in *Advances in Experimental Social Psychology* Vol. 9 eds Berkowitz L. Walster W. (New York, NY: Academic Press), 91–131.

[B52] Levine J. M. Moreland R. L. (1987). “Social comparison and outcome evaluation in group contexts,” in *Social Comparison, Social Justice, and Relative Deprivation: Theoretical, Empirical, and Policy Perspectives*, eds Masters J. C. Smith W. P. (Hillsdale, NJ: Lawrence Erlbaum Associates, Inc), 105–127.

[B53] Levine J. M. Moreland R. L. (1991). “Culture and socialization in work groups,” in *Perspectives on Socially Shared Cognition*, eds Resnick L. B. Levine J. M. Teasley S. D. (Washington, DC: American Psychological Association), 257–279. 10.1037/10096-011

[B54] Liao H. Liu D. Loi R. (2010). Looking at both sides of the social exchange coin: a social cognitive perspective on the joint effects of relationship quality and differentiation on creativity. *Acad. Manage. J.* 53 1090–1109. 10.5465/amj.2010.54533207

[B55] Liao H. Rupp D. E. (2005). The impact of justice climate and justice orientation on work outcomes: a cross-level multifoci framework. *J. Appl. Psychol.* 90 242–256. 10.1037/0021-9010.90.2.242 15769235

[B56] Lin X. Leung K. (2014). What signals does procedural justice climate convey? The roles of group status, and organizational benevolence and integrity. *J. Organ. Behav.* 35 464–488. 10.1002/job.1899

[B57] Lo S. Aryee S. (2003). Psychological contract breach in a Chinese context: an integrative approach. *J. Manage. Stud.* 40 1005–1020. 10.1111/1467-6486.00368

[B58] Mayer D. Nishii L. Schneider B. Goldstein H. (2007). The precursors and products of justice climates: group leader antecedents and employee attitudinal consequences. *Pers. Psychol.* 60 929–963. 10.1111/j.1744-6570.2007.00096.x

[B59] Morrison E. W. Robinson S. L. (1997). When employees feel betrayed: a model of how psychological contract violation develops. *Acad. Manage. Rev.* 22 226–256. 10.5465/amr.1997.9707180265

[B60] Mossholder K. W. Bennett N. Martin C. L. (1998). A multilevel analysis of procedural justice context. *J. Organ. Behav.* 19 131–141. 10.1002/(SICI)1099-1379(199803)19:2<131::AID-JOB878>3.0.CO;2-P 23623944

[B61] Naumann S. E. Bennett N. (2000). A case for procedural justice climate: development and test of a multilevel model. *Acad. Manage. J.* 43 881–889.

[B62] Organ D. W. (1988). *Organizational Citizenship Behavior: The Good Soldier Syndrome.* Lexington, MA: Lexington Books.

[B63] Ostroff C. Kinicki A. J. Tamkins M. M. (2003). “Organizational culture and climate,” in *Handbook of Psychology* Vol. 12 eds Borman W. C. Ilgen D. R. Klimoski R. J. (New York, NY: Wiley Online Library), 565–593.

[B64] Parker D. Reason J. T. Manstead A. S. R. Stradling S. G. (1995). Driving errors, driving violations and accident involvement. *Ergonomics* 38 1036–1048. 10.1080/00140139508925170 29105607

[B65] Peng A. Schaubroeck J. Li Y. (2014). Social exchange implications of own and coworkers’ experiences of supervisory abuse. *Acad. Manage. J.* 57 1385–1405. 10.5465/amj.2012.0080

[B66] Preacher K. J. Curran P. J. Bauer D. J. (2006). Computational tools for probing interactions in multiple linear regression, multilevel modeling, and latent curve analysis. *J. Educ. Behav. Statist.* 31 437–448. 10.3102/10769986031004437

[B67] Raudenbush S. W. Bryk A. S. Congdon R. (2004). *HLM 6 for Windows [Computer software].* Lincolnwood, IL: Scientific Software International.

[B68] Restubog S. L. D. Bordia P. Tang R. L. (2007). Behavioural outcomes of psychological contract breach in a non-western culture: the moderating role of equity sensitivity^∗^. *Br. J. Manage.* 18 376–386. 10.1111/j.1467-8551.2007.00531.x

[B69] Restubog S. L. D. Bordia P. Tang R. L. Krebs S. A. (2010). Investigating the moderating effects of leader-member exchange in the psychological contract breach-employee performance relationship: a test of two competing perspectives. *Br. J. Manage.* 21 422–437.

[B70] Restubog S. L. D. Zagenczyk T. J. Bordia P. Bordia S. Chapman G. J. (2015). If you wrong us, shall we not revenge? Moderating roles of self-control and perceived aggressive work culture in predicting responses to psychological contract breach. *J. Manage.* 41 1132–1154. 10.1177/0149206312443557

[B71] Robinson S. L. Morrison E. W. (2000). The development of psychological contract breach and violation: a longitudinal study. *J. Organ. Behav.* 21 525–546. 10.1002/1099-1379(200008)21:5<525::AID-JOB40>3.0.CO;2-T

[B72] Rosen C. C. Chang C. H. Johnson R. E. Levy P. E. (2009). Perceptions of the organizational context and psychological contract breach: assessing competing perspectives. *Organ. Behav Hum. Decis. Process.* 108 202–217. 10.1016/j.obhdp.2008.07.003

[B73] Rousseau D. M. (1995). *Psychological Contracts in Organizations: Understanding Written and Unwritten Agreements.* Thousand Oaks, CA: Sage Publications.

[B74] Rousseau D. M. Schalk R. (2000). *Psychological Contracts in Employment: Cross-National Perspectives.* Thousand Oaks, CA: Sage.

[B75] Salancik G. R. Pfeffer J. (1978). A social information processing approach to job attitudes and task design. *Admin. Sci. Quart.* 23 224–253. 10.2307/2392563 10307892

[B76] Schaubroeck J. Lam S. S. K. Cha S. E. (2007). Embracing transformational leadership: team values and the impact of leader behavior on team performance. *J. Appl. Psychol.* 92 1020–1030. 10.1037/0021-9010.92.4.1020 17638462

[B77] Skarlicki D. P. Latham G. P. (2006). Leadership training in organizational justice to increase citizenship behavior within a labor union: a replication. *Pers. Psychol.* 50 617–633. 10.1111/j.1744-6570.1997.tb00707.x

[B78] Snijders T. A. B. Bosker R. J. (2011). *Multilevel Analysis: An Introduction to Basic and Advanced Multilevel Modeling.* London: Sage Publications.

[B79] Tangirala S. Ramanujam R. (2008). Employee silence on critical work issues: the cross level effects of procedural justice climate. *Pers. Psychol.* 61 37–68. 10.1111/j.1744-6570.2008.00105.x

[B80] Thibaut J. W. Walker L. (1975). *Procedural Justice: A Psychological Analysis.* Hillsdale, MI: Erlbaum Associates.

[B81] Thomas D. C. Au K. Ravlin E. C. (2003). Cultural variation and the psychological contract. *J. Organ. Behav.* 24 451–471. 10.1002/job.209

[B82] Treviño L. K. Butterfield K. D. McCabe D. L. (1998). The ethical context in organizations: influences on employee attitudes and behaviors. *Bus. Ethics Quart.* 8 447–476. 10.2307/3857431

[B83] Turnley W. H. Bolino M. C. Lester S. W. Bloodgood J. M. (2003). The impact of psychological contract fulfillment on the performance of in-role and organizational citizenship behaviors. *J. Manage.* 29 187–206. 10.1177/014920630302900204

[B84] Turnley W. H. Feldman D. C. (1999). The impact of psychological contract violations on exit, voice, loyalty, and neglect. *Hum. Relat.* 52 895–922. 10.1177/001872679905200703

[B85] Tyler T. R. (1994). Psychological models of the justice motive: antecedents of distributive and procedural justice. *J. Pers. Soc. Psychol* 67 850–863. 10.1037/0022-3514.67.5.850

[B86] Tyler T. R. Blader S. L. (2000). *Cooperation in Groups: Procedural Justice, Social Identity, and Behavioral Engagement.* Philadelphia, PA: Psychology Press.

[B87] van Dijke M. De Cremer D. Langendijk G. Anderson C. (2018). Ranking low, feeling high: how hierarchical position and experienced power promote prosocial behavior in response to procedural justice. *J. Appl. Psychol.* 103 164–181. 10.1037/apl0000260 28933910

[B88] Van Dijke M. De Cremer D. Mayer D. M. Van Quaquebeke N. (2012). When does procedural fairness promote organizational citizenship behavior? Integrating empowering leadership types in relational justice models. *Organ. Behav. Hum. Decis. Process.* 117 235–248. 10.1016/j.obhdp.2011.10.006

[B89] Van Scotter J. R. Motowidlo S. J. (1996). Interpersonal facilitation and job dedication as separate facets of contextual performance. *J. Appl. Psychol.* 81 525–531. 10.1037/0021-9010.81.5.525

[B90] Vidyarthi P. R. Anand S. Liden R. C. (2014). Do emotionally perceptive leaders motivate higher employee performance? The moderating role of task interdependence and power distance. *Leadersh. Quart.* 25 232–244. 10.1016/j.leaqua.2013.08.003

[B91] Weick K. E. (1995). *Sensemaking in Organizations.* Thousand Oaks, CA: Sage.

[B92] West M. (2012). “The essence of high performance teams,” in *Ready for Change?: Transition Through Turbulence to Reformation and Transformation*, ed. Rathbone C. L. H. (London: Palgrave Macmillan), 111–127.

[B93] Williams L. J. Anderson S. E. (1991). Job satisfaction and organizational commitment as predictors of organizational citizenship and in-role behaviors. *J. Manage.* 17 601–617. 10.1177/014920639101700305

[B94] Yang J. Mossholder K. W. Peng T. K. (2007). Procedural justice climate and group power distance: an examination of cross-level interaction effects. *J. Appl. Psychol.* 92 681–692. 10.1037/0021-9010.92.3.681 17484550

[B95] Yang K. S. Yu A. B. Yeh M. H. (1989). “Chinese individual modernity and traditionality: construction definition and measurement [in Chinese],” in *Chinese Psychology and Behavior*, eds Yang K. S. Yu A. B. (Taipei: Laureat), 241–306.

[B96] Zhang K. Song L. J. Hackett R. D. Bycio P. (2006). Cultural boundary of expectancy theory-based performance management: a commentary on denisi and pritchard’s performance improvement model. *Manage. Organ. Rev.* 2 279–294. 10.1111/j.1740-8784.2006.00044.x

[B97] Zhao H. Wayne S. J. Glibkowski B. C. Bravo J. (2007). The impact of psychological breach on work-related outcomes: a meta-analysis. *Pers. Psychol.* 60 647–680. 10.1111/j.1744-6570.2007.00087.x

[B98] Zohar D. Hofmann D. (2012). “Organizational culture and climate,” in *Oxford Handbook of Industrial and Organizational Psychology*, ed. Kozlowski S. W. J. (Oxford: Oxford University Press), 643–666.

[B99] Zohar D. Luria G. (2005). A multilevel model of safety climate: cross-level relationships between organization and group-level climates. *J. Appl. Psychol.* 90 616–628. 10.1037/0021-9010.90.4.616 16060782

